# PLK1 gene suppresses cell invasion of undifferentiated thyroid carcinoma through the inhibition of CD44v6, MMP-2 and MMP-9

**DOI:** 10.3892/etm.2012.729

**Published:** 2012-09-28

**Authors:** XING-GUANG ZHANG, XIAO-FENG LU, XIU-MING JIAO, BIN CHEN, JIN-XIAO WU

**Affiliations:** Department of Endocrinology, The Military General Hospital of Beijing PLA, Beijing 100700, P.R. China

**Keywords:** polo-like kinase 1, anaplastic thyroid carcinoma, RNA interference, cell invasion, matrix metalloproteinase-2, matrix meta-lloproteinase-9, CD44v6

## Abstract

The aim of this study was to observe the regulatory action of the polo-like kinase 1 (PLK1) gene in the invasion of anaplastic thyroid carcinoma cells and investigate its mechanisms. The expression of the PLK1 protein in 36 patients with anaplastic thyroid carcinoma was detected by immunohistochemical staining. siRNA against PLK1 was designed, synthesized and transfected into ARO cells. The effects of PLK1 siRNA on cell invasion were detected by a soft agar colony formation assay and a Transwell chamber assay. The corresponding protein was detected using western blot analysis. The expression of PLK1 in anaplastic thyroid carcinoma samples (67.5±10.6%) was significantly higher compared to that in cancer-adjacent samples (0.65%±0.12%; P<0.01). The expression of PLK1 correlated with clinical stage, lymph node metastasis and prognosis of anaplastic thyroid. The number of cell clones was reduced in a dose-dependent manner with increasing levels of siRNA and the number of cells permeating through the filter membrane decreased following transfection with siRNA. The inhibition of PLK1 caused a significant decrease in CD44v6, matrix metalloproteinase (MMP)-2 and MMP-9 (0.36±0.08, 0.12±0.03, 0.25±0.06, respectively) compared to the non-transfected group (1.15±0.18, 1.21±0.20, 1.25±0.21, respectively; P<0.01). In conclusion, the expression of PLK1 was found to be increased in anaplastic thyroid carcinoma and was correlated with clinical stage, lymph node metastasis and prognosis. Additionaly, PLK1 siRNA was found to inhibit the invasion of anaplastic thyroid carcinoma cells. Therefore, CD44v6, MMP-2 and MMP-9 are likely to be involved in the regulation of cell invasion induced by PLK1.

## Introduction

Polo-like kinase 1 (PLK1) is a highly conserved serine/threonine kinase that plays an important role in mitosis (1). PLK1 is highly expressed in numerous tumor tissues or cells and is associated with clinical stage, metastasis and invasiveness, as well as with the prognosis of patients with tumors. PLK1 promotes tumor cell proliferation and cell transformation, thus, it is a new target for antitumor therapy (2). Thyroid carcinoma is a common malignancy of the endocrine system. There is no curative regimen for undifferentiated thyroid carcinoma, which is highly malignant and progresses rapidly. The PLK1 gene is known to play an important regulatory role in the cell proliferation of undifferentiated thyroid carcinoma. PLK1 RNA interference (RNAi) suppresses cell proliferation and induces apoptosis (3). However, the association between PLK1 and thyroid carcinoma cell invasiveness has yet to be adequately investigated. In the present study, we first examined the relationship among PLK1 expression and clinical stage and lymphatic metastasis in undifferentiated thyroid carcinoma tissue. The effect of PLK1 RNAi on the invasiveness of undifferentiated thyroid carcinoma ARO cells was then investigated. Additionally, we explored the possible mechanism behind CD44v6/matrix metalloproteinase (MMP)-2/MMP-9.

## Materials and methods

### Specimens

A total of 36 undifferentiated thyroid carcinoma specimens were obtained from 36 patients who underwent thyroidectomy between 2001 and 2007 at The Military General Hospital of Beijing PLA (Beijing, China). There were 26 males and 10 females, aged 21–72 (47.2±11.3) years. According to the clinical stage criteria set by the American Joint Commission for Cancer (AJCC), there were 10 cases at stage I, 11 cases at stage II, 8 cases at stage III and 7 cases at stage IV. Twenty-one cases were identified with lymphatic metastasis and 15 cases were identified without lymphatic metastasis. Of the patients included in this study, 20 patients had succumbed during the follow-up period of 5 years.

### Cell strain and antibodies

The undifferentiated human thyroid carcinoma cell line ARO was maintained in our laboratory. DMEM containing 10% fetal bovine serum was purchased from Gibco-BRL (Carlsbad, CA, USA). Goat anti-human PLK1, CD44v6, MMP-2 and MMP-9 antibodies were purchased from Santa Cruz Biotechnology Inc., (Santa Cruz, CA, USA). Horseradish peroxidase (HRP) conjugated rabbit anti-goat antibody was purchased from Boster Biotechnology Ltd. (Wuhan, China).

### Construction of PLK1 siRNA sequence and transfection of ARO cells

The PLK1 cDNA sequence was retrieved from GenBank, and three siRNA sequences (S1, S2, S3) (3) as well as a negative control sequence (Sn) were designed and synthesized (Table I). All sequences were synthesized by Takara (Dalian, China) and were verified by sequencing. The sequences (100 nM) were transfected into ARO cells using Oligofectamine™ (Invitrogen, Carlsbad, CA, USA). ARO cells were adjusted to 1×10^5^ cells/ml. The cells were divided into six groups: the blank group (ConB), void vector group (ConA), S1 transfection group (S1), S2 transfection group (S2), S3 transfection group (S3) and the Sn transfection group (Sn). The blank group was transfected with PBS and the void vector group with void vectors of the same concentration. The other treatments were the same among all groups. siRNA with the highest interference efficiency was used in the subsequent assay.

### Immunohistochemical detection of PLK1 protein expression

The surgical specimens were fixed with 10% formalin and embedded with paraffin. The sections were stained with an LSAB™ kit (Dako, Glostrup, Denmark). In brief, the sections were dewaxed conventionally to water, immersed in 200 ml citrate buffer, heated for 15 min and then transferred into a humid box. The sections were blocked for 10 min with 10% goat serum diluted with PBS at room temperature, incubated overnight with primary antibody (1:5,000) at 4°C and incubated for 30 min with biotin-conjugated secondary antibody (1:10,000) at 37°C in the humid box. This was followed by a 5 min incubation for color development, hematoxylin staining and mounting. Positive cells had brown- or yellow-stained nuclei or cytoplasm. Imag-Pro-Plus image analysis software was used to determine the area (%) of positive cells relative to the reference system.

### Western blot analysis of protein expression

Total cell protein was extracted by cell lysis with RIPA lysis buffer and 5-min centrifugation at 4°C and 10,000 rpm (centrifugation radius, 4 cm). The supernatants were harvested and protein concentration was determined using the BCA method. Protein (50 μg) was added with 2X loading buffer. After a 5-min denaturation at 100°C, proteins were subjected to SDS-PAGE and then transferred onto a nitrocellulose filter. The filter was incubated with specific primary and secondary antibodies, followed by enhanced chemiluminescence (ECL; Boster Biotechnology, Ltd.) and autoradiography. The resultant autoradiograms were subjected to grayscale analysis with BandScan software.

### Colony formation assay

Cells at the logarithmic phase were digested conventionally and suspended (1×10^3^ cells/ml). Agarose (5%) and culture medium were mixed at 1:9 and placed into culture dishes at room temperature. The cell suspension (1.5 ml) was added to 1.5 ml of 0.5% agarose, agitated and then added to the preprepared agarose dishes. Colony formation was observed after a 2-week culture at 37°C in air containing 5% CO_2_. The colony formation rate (%) was calculated using the equation: (number of colonies/number of cells inoculated) ×100%.

### In vitro invasion assay

The cell suspension was adjusted to 1×10^5^ cells/ml. The cell suspension (50 μl) was added to the upper chamber of Transwell (Chemicon, Temecula, CA, USA) and cultured for 24 h. The cells adhering to the interior of chamber were collected and fixed with 10% formalin, followed by Giemsa staining. Cells that had penetrated the membrane were counted.

### Statistical analysis

Data were expressed as the mean ± SD, and the two-sided Student’s t-test was performed using SPSS 16.0 software. P<0.05 was considered to indicate a statistically significant result.

## Results

### Correlation between PLK1 expression and clinical stage, metastasis and prognosis of thyroid carcinoma

**Figure 1:**
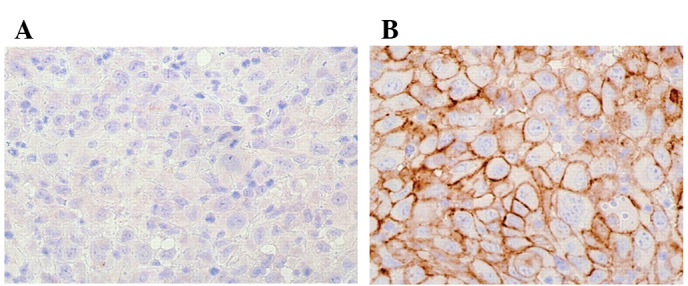
PLK1 protein expression upregulation in undifferentiated thyroid carcinoma tissue (SAB; magnification, x100). (A) Immunohistochemical study showed that PLK1 protein was expressed weakly in cancer-adjacent tissues. (B) A significantly stronger expression of PLK1 in undifferentiated thyroid carcinoma tissue is evident. PLK1, polo-like kinase 1.

Immunohistochemical investigation demonstrated that PLK1 protein was expressed weakly in cancer-adjacent tissues (0.65±0.12%). Moreover, the protein exhibited a significantly stronger expression in undifferentiated thyroid carcinoma samples (67.5±10.6%). Immunoreactive sites were mainly located in the cytoplasm and nuclei of follicular epithelia (Fig. 1). In addition, PLK1 expression correlated with clinical stage, lymphatic metastasis and prognosis of undifferentiated thyroid carcinoma (Table II).

### siRNA effect on PLK1 protein expression

**Figure 2:**
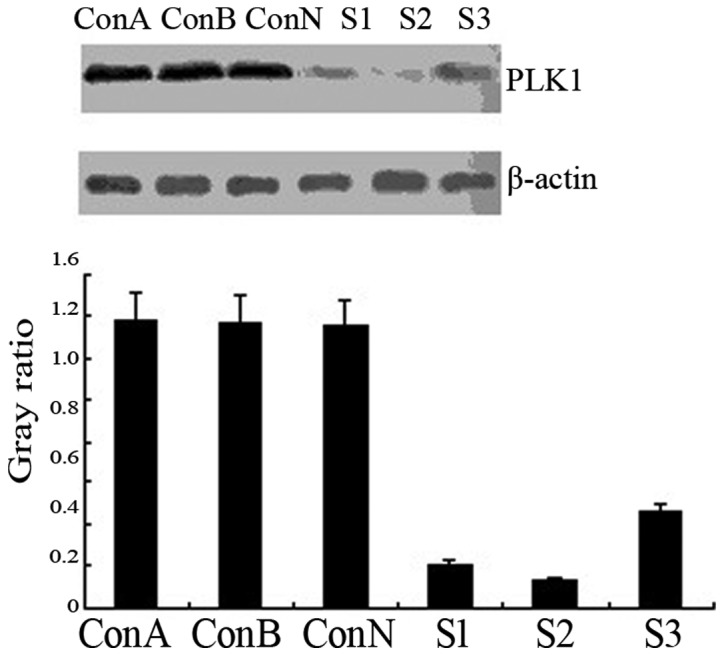
siRNA transfection of ARO cells silenced PLK1 gene specifically and suppressed PLK1 protein expression. PLK1, polo-like kinase 1.

Western blot analysis revealed that the levels of PLK1 protein expression were similarly high in the blank, negative control and void vector groups (P>0.05). Following siRNA transfection, PLK1 protein expression was significantly downregulated in the transfection groups (P<0.01), particularly in the S2 group (90.6% reduction vs. the control group; Fig. 2). The results demonstrated that the siRNA transfection of ARO cells silenced the PLK1 gene specifically and suppressed PLK1 protein expression.

### PLK1 siRNA effect on ARO cell anchorage-dependent growth

**Figure 3:**
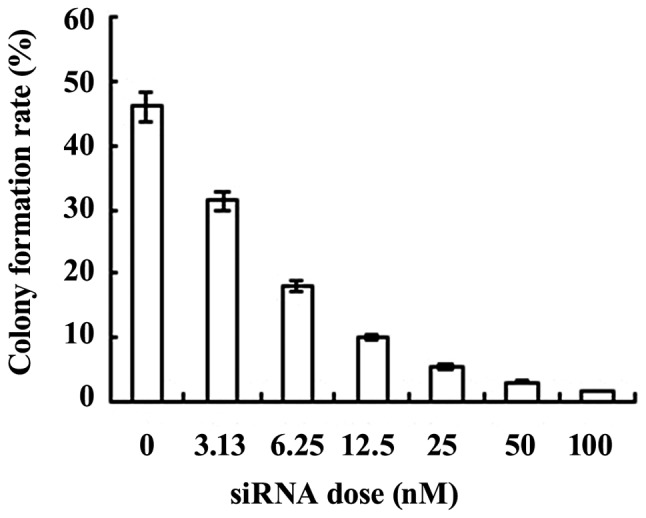
The colony formation rate decreased with increasing concentrations of S2 siRNA.

S2 exhibited the highest PLK1 interference efficiency. Thus, S2 was used for specific PLK1 interference. The colony formation assay showed that ARO cells formed colonies spontaneously in an *in vitro* culture system. The colony formation rate decreased with increasing concentrations of S2 siRNA (0, 3.125, 6.25, 12.5, 25, 50 and 100 nM; Fig. 3).

### Effect of PLK1 interference on ARO cell invasion

**Figure 4:**
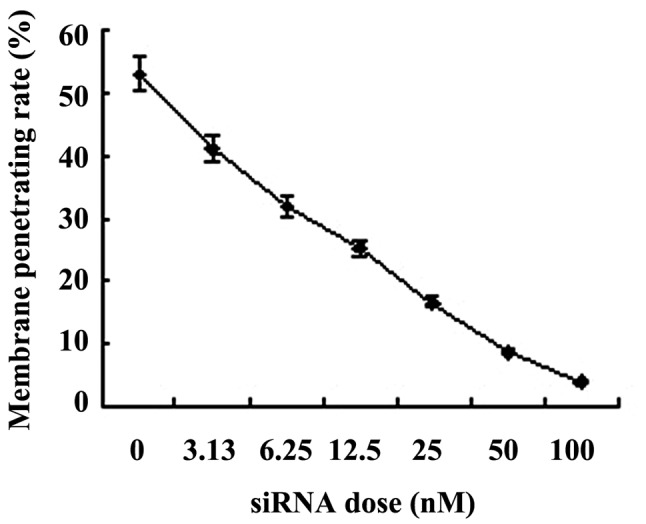
PLK1 interference decreased membrane-penetrating cells significantly in a siRNA concentration-dependent manner.

The results showed that PLK1 interference decreased membrane- penetrating cells significantly in a siRNA concentration- dependent manner (P<0.01; Fig. 4).

### Effect of PLK1 interference on CD44v6, MMP-2 and MMP-9 proteins

**Figure 5:**
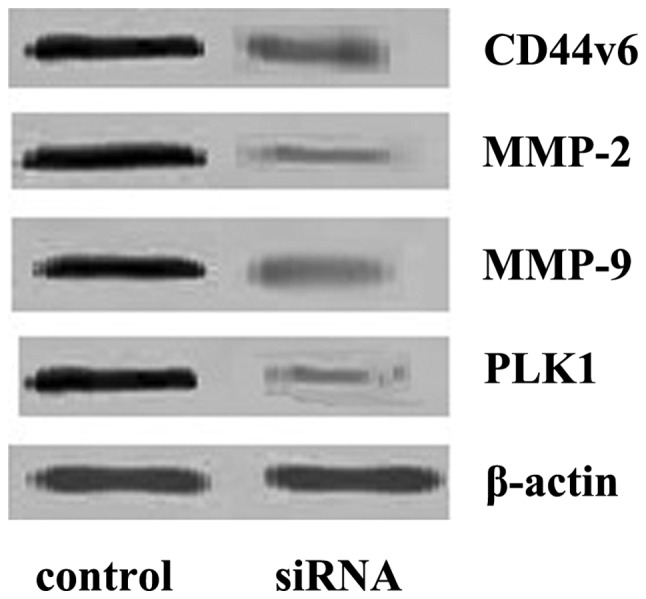
PLK1 interference decreased CD44v6, MMP-2 and MMP-9 proteins significantly when compared to the control group. PLK1, polo-like kinase 1.

The results showed that PLK1 interference decreased the level of CD44v6 protein significantly (0.36±0.08) when compared to the control group (1.15±0.18; P<0.01). MMP-2 and MMP-9 expression in the interference group (0.12±0.03, 0.25±0.06, respectively) decreased significantly when compared to the control group (1.21±0.20, 1.25±0.21; P<0.01; Fig. 5).

## Discussion

It has been demonstrated that the PLK1 gene plays an important role in the incidence and development of tumors. PLK1 expression is upregulated in a number of tumors including esophageal carcinoma (4,5), multiple myeloma (6), gynecological malignant tumors (7), skin cancer (8), liver cancer (9), gastric carcinoma (10) and cervical cancer (11). PLK1 expression status correlates with clinical stage, lymphatic metastasis and prognosis. The present study demonstrated that PLK1 protein expression is upregulated significantly in cancer tissues when compared with cancer-adjacent tissues, and that PLK1 expression correlated with clinical stage and lymphatic metastasis of thyroid carcinoma, as well as with the prognosis of patients. The higher the PLK1 expression levels, the higher the mortality rate of patients. This may be associated with the suppression of apoptosis and promotion of surviving activity by PLK1 (4). Salvatore *et al* (12) demonstrated that the PLK1 gene was highly expressed in undifferentiated thyroid carcinoma cells and therefore suppressed P53 and pRB genes. PLK1 RNAi led to cell cycle arrest. Therefore, PLK1 may become a molecular target for the treatment of undifferentiated thyroid carcinoma.

Since PLK1 is associated with the incidence, development and prognosis of tumors, the role of PLK1 activity suppression in tumor therapy and prognosis has become the focus of research. In recent years, RNAi technology has been widely used to silence PLK1 expression and test the role of PLK1 in the incidence, development and treatment of tumors. It was observed that the suppression of PLK1 activity decreased the survival rate of undifferentiated thyroid carcinoma cells significantly (13). Therefore, PLK1 gene therapy may be a promising treatment for undifferentiated thyroid carcinoma (14). The present study has shown that siRNA suppressed PLK1 efficiently.

To investigate the role of PLK1 in the invasion and metastasis of undifferentiated thyroid carcinoma cells, cell anchorage dependence and invasiveness were assessed in the present study via the specific RNAi of PLK1, colony formation assay and Transwell based *in vitro* invasion assay. Cell anchorage dependence refers to the fact that cells may avoid apoptosis and survive only by adhering to a specific matrix. By contrast, tumor cells are able to survive without adhering to a specific matrix, which is known as cell anchorage dependence. A colony formation assay may be used to assess tumor cell anchorage dependence and tumor malignancy (15). The invasiveness of cancer cells correlates positively with the number of colonies formed. The present study indicates that PLK1 siRNA suppressed ARO cell colony formation in soft agarose medium in a dose-dependent manner. This finding suggests that PLK1 interference suppresses the invasiveness of ARO cells. The metastasis and invasiveness of tumor cells are associated with the microenvironment and extracellular matrix (ECM) in which tumor cells grow. The Transwell model, which simulates the ECM is a reliable tool for assessing cell invasiveness (16). The present study has demonstrated that siRNA interference of PLK1 decreased membrane-penetrating cells significantly in a concentration-dependent manner.

Tumor invasion and metastasis is closely associated with the ability of tumor cells to induce the production of proteinases that degrade the ECM and basement membrane (17). A number of molecules are involved in regulating tumor cell invasion and metastasis. For instance, CD44v6 is an important member of the cell adhesion molecule CD44 family. CD44v6 regulates the ECM, enhances cell motility and suppresses tumor apoptosis. Therefore, it is crucial in tumor invasion and metastasis (18). The present study has shown that PLK1 interference suppressed ARO cell invasion and decreased CD44v6 activity, which supports the involvement of CD44v6 in tumor invasion and metastasis. MMPs are also associated with tumor invasion. MMP-2 (19,20) and MMP-9 (19,21–22) expression was upregulated in thyroid carcinoma and associated with lymphatic metastasis. Findings of the present study have shown that PLK1 suppression may inhibit MMP-2 and MMP-9 activity, thus suppressing cell invasion.

In conclusion, PLK1 expression was upregulated in undifferentiated thyroid carcinoma and correlated with clinical stage, lymphatic metastasis and tumor prognosis. PLK1 siRNA suppressed the cell invasion of undifferentiated thyroid carcinoma, and CD44v6, MMP-2 and MMP-9 contributed to the regulation of cell invasion of undifferentiated thyroid carcinoma via PLK1.

## Figures and Tables

**Table I:** PLK1 siRNA sequences.

siRNA	Sequences
S1	Sense, 5′-GAUUGUGCCUAAGUCUCUGTT-3′
	Antisense, 5′-CAGAGACUUAGGCACAAUCTT-3′
S2	Sense, 5′-UGAAGAUCUGGAGGUGAAATT-3′
	Antisense, 5′-CACCUCGAAACUGUGCUCUTT-3′
S3	Sense, 5′-UUCUCCGAACGUGUCACGUTT-3′
	Antisense, 5′-CACCUCGAAACUGUGCUCUTT-3′
Sn	Sense, 5′-UUCUCCGAACGUGUCACGUTT-3′
	Antisense, 5′-ACGUGACACGUUCGGGAATTT-3′

PLK1, polo-like kinase 1.

**Table II:** PLK1 expression correlated with the clinical stage, lymphatic metastasis and prognosis of undifferentiated thyroid carcinoma.

Clinical pathology	Case no.	PLK1 expression (mean ± SD%)	t	P-value
Gender				
M	26	63.2±11.3	0.20	0.83
F	10	64.1±12.4		
Age (years)				
<40	15	59.3±8.9	1.63	0.11
≥40	21	65.2±11.8		
Lymphatic metastasis				
Yes	15	46.5±7.8	6.91	0.00
No	21	71.3±13.6		
Clinical stage				
I, II	21	48.9±8.7	6.06	0.00
III, IV	15	69.2±11.4		
Prognosis				
Survived	16	36.5±4.9	9.52	0.00
Mortalities	20	71.3±15.4		

PLK1, polo-like kinase 1; M, male; F, female.
